# Draft Genome Sequence of *Frankia* sp. Strain QA3, a Nitrogen-Fixing Actinobacterium Isolated from the Root Nodule of *Alnus nitida*

**DOI:** 10.1128/genomeA.00103-13

**Published:** 2013-03-21

**Authors:** Arnab Sen, Nicholas Beauchemin, David Bruce, Patrick Chain, Amy Chen, Karen Walston Davenport, Shweta Deshpande, Chris Detter, Teal Furnholm, Faten Ghodbhane-Gtari, Lynne Goodwin, Maher Gtari, Cliff Han, James Han, Marcel Huntemann, Natalia Ivanova, Nikos Kyrpides, Miriam L. Land, Victor Markowitz, Kostas Mavrommatis, Matt Nolan, Imen Nouioui, Ioanna Pagani, Amrita Pati, Sam Pitluck, Catarina L. Santos, Saubashya Sur, Ernest Szeto, Fernando Tavares, Hazuki Teshima, Subarna Thakur, Luis Wall, Tanja Woyke, Jessie Wishart, Louis S. Tisa

**Affiliations:** b University of New Hampshire, Durham, New Hampshire, USA; a University of North Bengal, Siliguri, India; e University of Tunis-El Manar, Tunis, Tunisia; i University of Quilmes, Quilmes, Argentina; j Oregon State University, Corvallis, Oregon, USA; d DOE Joint Genome Institute, Walnut Creek, California, USA; c Los Alamos National Laboratory, Los Alamos, New Mexico, USA; f Oak Ridge National Laboratory, Oak Ridge, Tennessee, USA; g IBMC - Instituto de Biologia Celular e Molecular, Porto, Portugal; h CIBIO, Centro de Investigação em Biodiversidade e Recursos Genéticos, Vairão, Portugal

## Abstract

Members of the actinomycete genus *Frankia* form a nitrogen-fixing symbiosis with 8 different families of actinorhizal plants. We report a high-quality draft genome sequence for *Frankia* sp. strain QA3, a nitrogen-fixing actinobacterium isolated from root nodules of *Alnus nitida.*

## GENOME ANNOUNCEMENT

The genus *Frankia* consists of filamentous actinobacteria that form nitrogen-fixing symbiotic associations with a wide variety of woody angiosperms termed actinorhizal plants (1–3). The symbiosis allows actinorhizal plants to colonize harsh environmental terrains under diverse ecological conditions. Phylogenetic analysis based on several criteria, including the 16S rRNA gene (4), *glnII* (5, 6), and the 16S-23S rRNA intertranscribed spacer region (7), has identified four distinct clusters among the *Frankia* strains. Genomes for representatives from each of these clusters have been sequenced (8–10) and have provided vital baseline information for genomic approaches toward understanding these novel bacteria.

Members of cluster I *Frankia* strains are known to associate with *Betulaceae, Myricaceae*, and *Casuarinaceae* plants (except *Gymnostoma*) and include both narrow-host-range and mid-host-range symbionts. Since only two members of cluster I have been sequenced, strains ACN14a and CcI3 (8), another cluster I strain was sequenced to increase our understanding of this *Frankia* group and its host plant range. *Frankia* sp. strain QA3 was chosen for sequencing as another cluster I representative with mid-host-range properties. The strain was isolated from root nodules of *Alnus nitida* collected at an elevation of 1,240 m in the mountainous region of Bahrin, District Swat, Pakistan (11). *Frankia* sp. strain QA3 is also resistant to elevated levels of toxic heavy metals (12) and has potential applications in bioremediation. Strain QA3 was sequenced to provide information about the potential ecological roles of the *Frankia* strains and their interaction with actinorhizal plants.

The draft genome of *Frankia* sp. strain QA3 was generated at the Department of Energy (DOE) Joint Genome Institute (JGI) using a combination of 454-GS-FLX-Titanium (13) and Illumina GAii (14) techniques. A standard 454 titanium standard library, which generated 261,792 reads, a paired-end 454 library with an average insert size of 8 kb, which generated 728,635 reads totaling 258.7 Mb of 454 data, and an Illumina GAii shotgun library, which generated 116,789,226 reads totaling 8,876 Mb were created. All techniques for DNA isolation, library construction, and sequencing were performed according to JGI standards and protocols (http://www.jgi.doe.gov). The 454 Titanium standard data and the 454 paired-end data were assembled together with Newbler, version 2.3 (13), and resulted in 177.9 Mb of 454 draft data, which provided an average 23.4× coverage of the genome. The Illumina sequencing data were assembled with Velvet, version 1.0.13 (15), and the resulting 8,345.8 Mb of Illumina draft data provided an average 1,098.1× coverage of the genome. For finishing, the gaps and misassemblies were resolved by editing in Consed, by PCR, and by bubble PCR primer walks.

The high-quality draft genome of *Frankia* QA3 was resolved to 1 scaffold containing 121 contigs consisting of 7,590,853 bp with a G+C content of 72.6%, 6,493 candidate protein-encoding genes, 46 tRNA genes, and 3 rRNA regions.

### Nucleotide sequence accession number.

The whole draft genome sequences of *Frankia* strain QA3 have been deposited in GenBank with accession number AJWA00000000.
